# etymologia: Sapovirus [sap′ o-vi′′ rәs]

**DOI:** 10.3201/eid1407.071045

**Published:** 2008-07

Formerly Sapporo-like virus after Sapporo, Japan, where first recognized during an outbreak in an orphanage in 1977. A genus of viruses of the family Caliciviridae, they cause self-limited, acute foodborne gastroenteritis. Morphologically similar viruses were detected in a subsequent series of outbreaks in the same institution between 1977 and 1982. Sapoviruses play an important role in outbreaks of gastroenteritis in infants and have recently been found to infect adults. 

